# The expression of aldehyde dehydrogenase 1 (ALDH1) in ovarian carcinomas and its clinicopathological associations: a retrospective study

**DOI:** 10.1186/s12885-015-1513-5

**Published:** 2015-07-07

**Authors:** Ruixia Huang, Xiaoran Li, Ruth Holm, Claes G. Trope, Jahn M. Nesland, Zhenhe Suo

**Affiliations:** 1 Departments of Pathology, The Norwegian Radium Hospital, Oslo University Hospital, Ullernchausseen 70, 0379 Oslo, Norway; 2 Departments of Pathology, Institute of Clinical Medicine, Faculty of Medicine, University of Oslo, Oslo, Norway; 3 Departments of Gynaecology, The Norwegian Radium Hospital, Oslo University Hospital, Oslo, Norway; 4 Departments of Gynaecology, Institute of Clinical Medicine, Faculty of Medicine, University of Oslo, Oslo, Norway

**Keywords:** Ovarian carcinoma, Immunohistochemistry, ALDH1, Cancer stem cells, Stromal cells, Tumor microenvironment

## Abstract

**Background:**

Aldehyde dehydrogenase 1 (ALDH1) is widely used as a specific cancer stem cell marker in a variety of cancers, and may become a promising target for cancer therapy. However, the role of its expression in tumor cells and the microenvironment in different cancers is still controversial.

**Methods:**

To clarify the clinicopathological effect of ALDH1 expression in ovarian carcinoma, a series of 248 cases of paraffin-embedded formalin fixed ovarian carcinoma tissues with long term follow-up information were studied by immunohistochemistry.

**Results:**

The immunostaining of ALDH1was variably detected in both tumor cells and the stromal cells, although the staining in tumor cells was not as strong as that in stromal cells. Statistical analyses showed that high ALDH1 expression in tumor cells was significantly associated with histological subtypes, early FIGO stage, well differentiation grade and better survival probability (*p* < 0.05). The expression of ALDH1 in the stromal cells had no clinicopathological associations in the present study (*p* > 0.05).

**Conclusioms:**

High expression of cancer stem cell marker ALDH1 in ovarian carcinoma cells may thus portend a favorable prognosis, but its expression in tumor microenvironment may have no role in tumor behavior of ovarian carcinomas. More studies are warranted to find out the mechanisms for this.

## Background

Ovarian carcinoma remains the most mortality in gynecologic tumors [1]. There are 225,000 new cases diagnosed and 140,000 deaths of ovarian carcinoma annually worldwide [1]. The standard treatment remains surgery followed by platinum-based chemotherapy [2]. Acquired drug resistance and cancer recurrence become the main hurdles for ovarian carcinoma treatment currently [3]. As a result, new reagents targeting the chemo-resistant cells are needed.

Aldehyde dehydrogenases (ALDH) are a group of enzymes that catalyse dehydrogenation of aldehydes to their corresponding carboxylic acids. To date, nineteen ALDH genes which encode several isozymes have been identified in human genome. Aldehyde dehydrogenase 1 (ALDH1) gene encodes a cytosolic isoform localized in the cytoplasm and ALDH2 gene encodes a mitochondrial isoform located in mitochondrial matrix. Nevertheless, ALDH1 in human is not limited to the metabolic enzyme. It should be noted that ALDH1 is involved in regulating cell differentiation [4, 6], proliferation and motility [6, 7]. Its regulation role in stem cells is particularly through the retinoid signaling pathway [8, 9]. It is also reported that inhibition of ALDH1-mediated retinoid signaling impairs human fetal islet cell differentiation and survival [5]. ALDH1 may contribute to tumor initiation and chemoresistance [10]. In addition, it is regarded as a cancer stem cell (CSC) marker in a variety of cancers [11], including ovarian carcinoma [12, 13], lung cancer [14, 15], rectal cancer [16] and others [17, 18]. CSCs are a subpopulation of cancer cells which have the properties of self-renewal and tumorigenicity, and thus may play a key role in cancer metastasis, chemoresistance and relapse. Therapeutic modalities targeting CSCs are becoming a hot topic in recent years to prevent cancer relapse and vastly improve cancer survival probability [19]. Targeting CSC specific markers is one of the most important and easily achievable ways to identify putative CSCs. ALDH1, as a largely used stem cell marker in recent CSC studies, is mostly regarded as a poor prognostic factor in a variety of cancers [17, 18, 20, 21].

However, it remains debatable whether ALDH1 as a single marker can be sufficient to identify CSCs [22]. Futhermore, different isoforms of ALDH1 may serve variable roles in CSCs [23]. To date, the predictive role of ALDH1 in ovarian carcinoma cells and stromal cells are still obscure. While Chang et, al. found it was significantly associated with favorable clinical outcomes and better survivals in 442 cases of primary ovarian carcinoma patients [24], others insisted that it is an unfavorable prognostic factor in ovarian carcinomas [20, 21].

To further understand the prognositic role of ALDH1 in ovarian carcinoma cells and the stromal cells, we randomly enrolled 248 cases of primary ovarian carcinoma and investigated the expression of ALDH1 in these tissues by immunohistochemistry (IHC). The staining of ALDH1 in both carcinoma cells and stromal cells were evaluated and their associations with clinicalpathological parameters were analyzed by SPSS software.

## Methods

### Ethics statement

This study was approved by The Regional Committee for Medical Research Ethics South of Norway (S-06277a), The Social- and Health Directorate (06/3280) and The Data Inspectorate (06/5345).

### Clinical samples

Two-hundred and forty-eight surgically removed ovarian carcinoma samples were randomly enrolled in this study. All patients were diagnosed and operated at The Norwegian Radium Hospital, Oslo University Hospital during 1983 to 2000. The ages of the patients at diagnosis range from 19 to 89 years, with a median of 58 years. The patients were followed up until January 1st 2012. All the patients were clinically staged by the criteria of International Federation of Gynecology and Obstetrics (FIGO) stage [25]. The primary tumors were histologically graded as well, moderately and poorly differentiated according to WHO recommendations by two of the authors (J.M. and Z.S.) [26]. Disease progression was determined based on the definitions outlined by the Gynecologic Cancer Intergroup [27].

### IHC

Three-micrometres sections made from formalin-fixed paraffin embedded tissues were immunostained using Dako Envision™ FLEX+ system (K8012; Dako, Glostrup, Denmark) and the Dako Autostainer. Paraffin sections were deparaffinized and epitopes unmasked in PT-link with low pH target retrieval solution (Dako), and then blocked with peroxidase blocking (Dako) for 5 min. The slides were incubated at 4 °C overnight with mouse anti-human ALDH1 antibody (1: 3000, 83 ng IgG_1_/ml, Clone 44, Lot. No. 03817, BD Transduction Laboratories™), followed up by incubation with mouse linker for 15 min and HRP for 30 min at room temperature. Slides were then stained with 3, 3′-diaminobenzidine tetrahydrochloride (DAB) for 10 min and counter-stained with hematoxylin, dehydrated, and mounted in Richard-Allan Scientific Cyto seal XYL (Thermo Scientific, Waltham, MA, USA). Known ALDH1-positive human vulvar squamous cell carcinoma slide [28] was used as positive control. Mouse myeloma protein of the same subclass and concentration as the primary mouse anti-ALDH1 antibody was used for negative control.

### IHC scoring system

Allred scoring system [29, 30] was used for evaluating ALDH expression levels in ovarian carcinoma tissues. The ovarian carcinoma cells and the stromal cells were scored separately. The intensity of the immunohistochemical staining was scaled by 0 to 3 and the percentage of immunostaining cells was scaled by 0 to 5 (Table 1). The sum of intensity score and percentage score was seen as total score, which ranged from 0 to 8. The slide was regarded as ALDH negative, low expression and high expression when the total score is 0, 1 to 6 and 7 to 8, respectively. Examination of immunostaining was performed by two independent observers (RHuang and ZS), and all the cases were verified by another pathologist (JMN).

**Table 1:** The criteria of Allred scoring system used for evaluating ALDH1 expression in the ovarian carcinoma cells and the stromal cells in our study

1. The criteria of intensity scoring system						
Intensity Score	0	1	2	3		
	Negative	Weak	Moderate	Strikingly positive at low magnitude		
2. The criteria of percentage scoring system						
Percentage Score	0	1	2	3	4	5
	0	<1 %	1–10 %	11–33 %	34–66 %	67–100 %
3						
Total Score^a^	0		1–6		7–8	
	Negative		Low		High	

^a^The total score was obtained by adding the percentage score to intensity score. It ranges from 0 to 8

### Statistical analyses

SPSS software (version 18.0) was used for survival analysis and the analyses of the associations between ALDH1 expression and the clinical outcomes. Associations between categorical variables were assessed by Chi-square tests (Pearson and linear-by-linear as appropriate). Survival analysis was performed using the Kaplan-Meier method, and groups were compared with log-rank tests. Multivariate analysis was performed using Cox Regression method. Patients alive on the last follow-up date without recurrence were censored. For all the analyses, associations were considered to be significant if the *p* value was < 0.05.

## Results

### ALDH1 was variably detected in clinical ovarian carcinoma samples

**Fig. 1:**
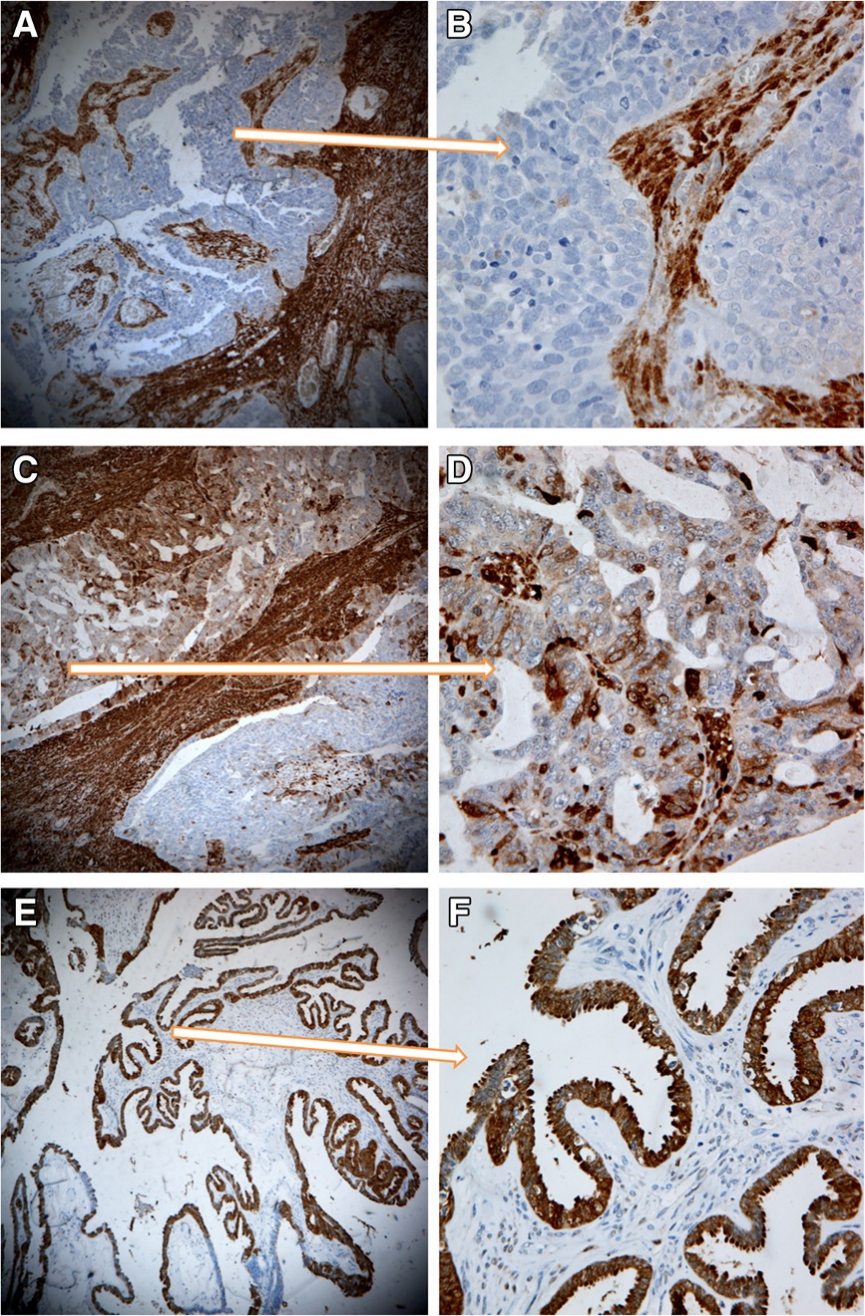
ALDH1 expression in ovarian carcinoma cells and stromal cells. **a** A poor differentiated ovarian carcinoma showed negative immunostaining in tumor cells for ALDH1, while most of the stromal cells surrounding tumor cells were strongly positive (100×). **b** High magnitude was used in the same case in A, showing clear negative tumor cells and positive stromal cells (400×). **c** Variable expression levels of ALDH1 in tumor cells were displayed in a moderately differentiated ovarian carcinoma. The stromal cells were all positive (100×). **d** A part of figure (**b**) was enlarged, showing weakly positive staining in most of the tumor cells and the staining was limited to the membrane and the cytoplasm (400×). **e** Tumor cells in a well differentiated ovarian carcinoma were all strongly positive while the stromal cells were negative (100×). **f** High magnitude of a part of figure (E) displayed strongly positive staining for ALDH1 in most of the epithelial tumor cells/hyperplasia epithelial cells (400×)

Immunoreactive ALDH1 was variably detected in the ovarian carcinoma cells and the stromal cells in all the ovarian primary tumor samples (Fig. 1). Endothelial cells of blood vessel were always positive for ALDH1. The immunostaining was limited to cytoplasm and cell membrane. Out of the total 248 samples, 98 cases were negative in tumor cells for ALDH1, and 111 cases had a low expression level and 39 cases had a high expression level (Table 2). Generally, the tumor cells from well-differentiated carcinomas tended to highly expressed ALDH1 and those from poor-differentiated carcinomas tended to express ALDH1 lowly. Compared with the tumor cells, ALDH1 expression in the stromal cells was generally rather strong. The numbers of negative, low expression and high expression of ALDH1 in the stromal cells were 13 cases, 61 cases and 174 cases, respectively (Table 3).

**Table 2:** The associations of ALDH1 expression levels in ovarian carcinoma cells and the clinical and pathologic characteristics

		ALDH1 IHC score in tumor cells			*p*-value
		1 (negative)	2 (low)	3 (high)	
Number	Total *N*	98	111	39	
Age (years old):					
≤39	16	5 (31.3 %)	6 (37.5 %)	5 (31.3 %)	
40–49	41	20 (48.8 %)	15 (36.6 %)	6 (14.6 %)	0.940
50–59	64	20 (31.3 %)	34 (53.1 %)	10 (15.6 %)	
60–69	72	28 (38.9 %)	35 (48.6 %)	9 (12.5 %)	
≥70	39	13 (33.3 %)	18 (46.2 %)	8 (20.5 %)	
missing	16				
Histological subtype:					
Serous carcinoma	163	67 (41.1 %)	80 (49.1 %)	16 (9.8 %)	
Mucinous carcinoma	18	4 (22.2 %)	3 (16.7 %)	11 (61.1 %)	<0.001
Endometrioid carcinoma	19	4 (21.1 %)	9 (47.4 %)	6 (31.6 %)	
Clear cell carcinoma	11	6 (54.5 %)	4 (36.4 %)	1 (9.1 %)	
Mixed epithelial tumor	11	2 (18.2 %)	6 (54.5 %)	3 (27.3 %)	
Undifferentiated tumor	5	3 (60.0 %)	1 (20.0 %)	1 (20.0 %)	
Unclassified tumor and others	21				
FIGO Stage:					
I	28	7 (25.0 %)	11 (39.3 %)	10 (35.7 %)	
II	18	8 (44.4 %)	6 (33.3 %)	4 (22.2 %)	
III	117	47 (40.2 %)	55 (47.0 %)	15 (12.8 %)	0.032
IV	79	31 (38.2 %)	38 (48.1 %)	10 (12.7 %)	
Not staged or missing	6				
Histological Grade:					
Well	19	4 (21.1 %)	9 (47.4 %)	6 (31.6 %)	
Moderate	62	20 (32.3 %)	35 (56.5 %)	7 (11.3 %)	0.012
Poor	133	58 (43.6 %)	60 (45.1 %)	15 (11.3 %)	
Not graded or missing	34

**Table 3:** The associations of ALDH1 expression levels in stromal cells and the clinical and pathologic characteristics

		ALDH1 IHC score in stromal cells			*p*-value
		1 (negative)	2 (low)	3 (high)	
Number	Total *N*	13	61	174	
Age (years old):					
≤39	16	0 (.0 %)	5 (31.3 %)	11 (68.8 %)	
40–49	41	3 (7.3 %)	10 (24.4 %)	28 (68.3 %)	0.360
50–59	64	2 (3.1 %)	19 (29.7 %)	43 (67.2 %)	
60–69	72	6 (8.3 %)	15 (20.8 %)	51 (70.8 %)	
≥70	39	1 (2.6 %)	6 (15.4 %)	32 (82.1 %)	
missing	16				
Histological subtype:					
Serous carcinoma	163	6 (3.7 %)	36 (22.1 %)	121 (74.2 %)	
Mucinous carcinoma	18	3 (16.7 %)	6 (33.3 %)	9 (50.0 %)	0.077
Endometrioid carcinoma	19	0 (.0 %)	7 (36.8 %)	12 (63.2 %)	
Clear cell carcinoma	11	1 (9.1 %)	1 (9.1 %)	9 (81.8 %)	
Mixed epithelial tumor	11	0 (.0 %)	4 (36.4 %)	7 (63.6 %)	
Undifferentiated tumor	5	1 (20.0 %)	1 (20.0 %)	3 (60.0 %)	
Unclassified tumor and others	21				
FIGO stage:					
I	28	2 (7.1 %)	8 (28.6 %)	18 (64.3 %)	
II	18	0 (.0 %)	10 (55.6 %)	8 (44.4 %)	
III	117	6 (5.1 %)	27 (23.1 %)	84 (71.8 %)	0.161
IV	79	5 (6.3 %)	14 (17.7 %)	60 (75.9 %)	
Not staged or missing	6				
Histological grade:					
Well	19	1 (5.3 %)	9 (47.4 %)	9 (47.4 %)	
Moderate	62	2 (3.2 %)	11 (17.7 %)	49 (79.0 %)	0.655
Poor	133	7 (5.3 %)	34 (25.6 %)	92 (69.2 %)	
Not graded or missing	34				

### ALDH1 expression was not associated with the ages

The ages at diagnosis were divided into five groups for the association analyses: ≤ 39, 40–49, 50–59, 60–69 and ≥ 70 years. The expression of ALDH1 in ovarian carcinoma cells was not significantly different (*p* > 0.05, linear by linear association). There was no significant difference between each age group for ALDH1 expression in the stromal cells as well (*p* > 0.05, linear by linear association).

### ALDH1 expression in ovarian carcinoma cells was associated with histological subtype

Ovarian carcinoma patients involved in our study were diagnosed and verified as several subtypes by histology: serous carcinoma, mucinous carcinoma, endometrioid carcinoma, clear cell carcinoma, mixed epithelial tumor, undifferentiated tumor and others. ALDH1 expression in tumor cells tended to be negative or low in serous carcinoma and clear cell carcinoma. There was significant difference for the ALDH1 expression levels in tumor cells between each histological subtypes, highest in mucinous carcinoma (*p* < 0.001, Pearson Chi-Square test, Table 2). However, the ALDH1 expression in stromal cells had no significant differences between each histological group (*p* > 0.05, Pearson Chi-Square test, Table 3).

### ALDH1 expression in ovarian carcinoma cells was associated with early FIGO stage

FIGO stage, as the most popular clinically used stage criteria is an important prognostic predictor in ovarian carcinomas [25]. High expression of ALDH1 in ovarian carcinoma cells was significantly associated with early FIGO stage (*p* < 0.05, linear by linear association, Table 2). The percentage of high ALDH1 expression in ovarian carcinoma cells distributes largely in FIGO stage I (35.7 %) and II (22.2 %) than FIGO stage III (12.8 %) and IV (12.7 %). However, no significant association between ALDH1 expression in tumor stromal cells and FIGO stage was discoverd (*p* > 0.05, linear by linear association, Table 3).

### ALDH1 expression in ovarian carcinoma cells was associated with well differentiation grade

Differentiation grade stands for the malignant potential in tumors. Well differentiated carcinomas are regarded as low malignant potential and conversely poor differentiated carcinomas as high malignant potential in ovarian carcinomas [26]. In the well differentiated carcinoma group the percentage of ALDH1 high expression cases in tumor cells (31.6 %) was much higher than the percentage in the moderate differentiation group (11.3 %) and the poor differentiation group (11.3 %), which was in accordance with our general finding when evaluating slides. The association between ALDH1 expression in tumor cells and the differentiation grade was significant (*p* < 0.05, linear by linear association, Table 2). Nevertheless, the ALDH1 expression in the stromal cells was not associated with the differentiation grade (*p* > 0.05, linear by linear association, Table 3).

### ALDH1 expression in ovarian carcinoma cells was associated with better survivals

There were totally 232 valid cases with full information for the analyses of overall survival (OS) and progression free survival (PFS). The median OS was 1.480 years with 95 % confidence interval (CI) of 1.076 years to 1.884 years. The median PFS was 0.650 year and the 95 % CI was 0.507 year to 0.793 year.

**Fig. 2:**
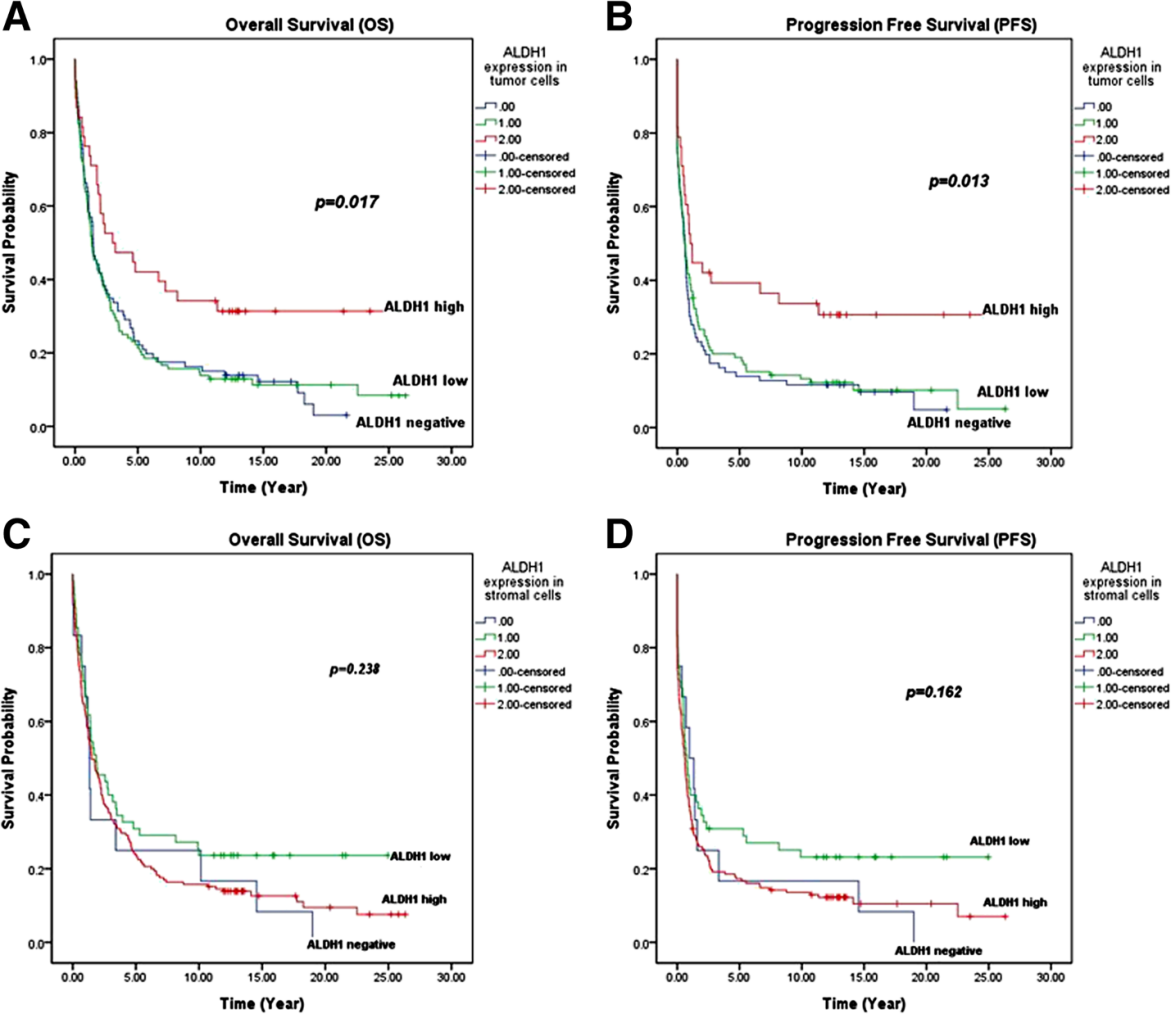
Survival probabilities in different ALDH1 expression groups. **a** Survival plot disclosed that ALDH1 high expression in tumor cells in ovarian carcinomas had a better OS than the low expression and Negative groups (*p* < 0.05, Kaplan-Meier). **b** High expression level of ALDH1 in tumor cells in ovarian carcinomas was significantly associated with high PFS probabilities (*p* < 0.05, Kaplan-Meier). **c** The expression level of ALDH1 in the stromal cells in ovarian carcinoma had no significant association with OS probabilities (*p* > 0.05, Kaplan-Meier). **d** No statistical association was found between the expression of ALDH1 in the stromal cells of ovarian carcinoma and the PFS probabilities (*p* > 0.05, Kaplan-Meier).

In the 232 patients, ALDH1 expression in tumor cells was negative in 86 cases, low in 108 cases and high in 38 cases. The median OSs in the ALDH1 tumor-negative group, tumor low-expression group and tumor high-expression group were 1.440 years, 1.330 years and 3.000 years, respectively. The median PFSs in the above three groups were 0.590 year, 0.580 year and 1.050 years, respectively. Furthermore, ALDH1 expression in ovarian carcinoma cells was significantly associated with better OS and PFS by statistical analyses (*p* < 0.05, Kaplan-Meier method, Fig. 2a, b).

Out of the 232 valid cases for survival analyses, ALDH1 expression in stromal cells was negative, low and high in 12 cases, 55 cases and 165 cases, respectively. The median OSs in the ALDH1 stromal-negative group, stromal-low group and stromal-high group were 1.320 years, 1.850 years and 1.480 years, respectively. The median PFSs in the above three groups were 0.980 year, 0.760 year and 0.610 year, respectively. ALDH1 expression in the stromal cells was thus not significantly associated with OS and PFS by statistical analyses (*p* > 0.05, Kaplan-Meier method, Fig. 2 c, d).

### ALDH1 expression in tumor cells is not independent risk factor for overall survival

Multivariate analysis was performed using Cox Regression method based on the above clinicopathological parameters and ALDH1 expression in tumor cells and stromal cells (Table 4). Age at diagnosis, FIGO stage and differentiation grade are independent risk factor for overall survival in ovarian carcinomas (*p* < 0.05), while other variables including histological subtypes, ALDH1 expression in tumor cells and ALDH1 expression in stromal cells does not contribute to overall survival independently (*p* > 0.05).

**Table 4:** Multivariate analysis of overall survival in a total of 232 valid ovarian carcinoma patients

Factors	HR^a^	95 % CI^b^	*p*-value
ALDH1 tumor^c^	0.913	0.735–1.134	0.412
ALDH1 stromal^c^	1.008	0.764–1.331	0.913
Age group^d^	1.193	1.048–1.357	0.007
Differentiation grade^e^	1.377	1.075–1.763	0.011
FIGO stage^f^	1.676	1.356–2.071	<0.001
Histological subtype^g^	0.966	0.879–1.062	0.472

^a^*HR* Hazard ratio; ^b^95 % CI, 95 % confidence interval; ^c^ALDH1 expression including negative group, low expression group and high expression group; ^d^Age ≤ 39 years versus 40–49 years versus 50–59 years versus 60–69 years versus ≥70 years; ^e^well differentiation versus moderate differentiation versus poor differentiation; ^f^FIGO stage I versus FIGO stage II versus FIGO stage III versus FIGO stage IV; ^g^Serous carcinoma versus mucinous carcinoma versus endometrioid carcinoma versus clear cell carcinoma versus mixed epithelial tumor versus undifferenciated tumor

## Discussion

Ovarian carcinoma is actually one of the most chemosensitive solid malignancies [31], but it is still the most lethal gynecologic malignancy worldwide. Although most ovarian carcinoma cells are initially sensitive to chemotherapy, there is always a small population cells that always survives and initiates new tumors which causes recurrence [31]. The verification of tumor heterogeneity further enhances the hypothesis of CSCs. Compelling evidence has shown that ovarian carcinomas with enriched CSCs exhibit aggressive features *in vitro* and predict poor outcomes in patients [21, 32, 33].

Theoretically a high proportion of CSCs in tumor should be correlated with poor prognosis. However, the CSC markers are not always universal in a given tumor. The candidates raised up to characterize ovarian CSCs include CD44, epithelial cell adhesion molecule (EpCAM), CD133, CD117, CD90 (Thy-1), CD24, ABCG2, LY6A, AGR5 and ALDH1, etc. [34, 35]. However, it still remains challenging to identify one single marker or several combined markers to specifically identify all the CSCs in ovarian tumor, and the exact roles of these ‘stemness’ related markers, are still poorly understood due to either a current lack of understanding of the biological functions of the markers, or frequently the lack of information correlating the varied isoforms, splicing variants or substrates to stem cell function [34, 35].

ALDH1 is initially found as a cytosolic isozyme located in the cytoplasm, mainly functioning as the second enzyme after alcohol dehydrogenase in the major pathway of alcohol metabolism in liver. In recent years, soon after the rise of CSC theory, some specific CSC markers were being discovered to identify putative CSCs, and ALDH1 is becoming to act as a CSC marker in the CSC studies of variable tumor types *in vitro* and *in vivo*. Epithelial-to-mesenchymal transition (EMT) is an important driver of tumor invasion and metastasis, which may be a feature of CSC. Compared to ALDH1(−) EMT cells, only ALDH1(+) EMT cells had the ability to initiate a new epithelial tumor [36]. Both EMT and other CSC properties of ALDH1(+) lung CSCs can be repressed through Fibulin3 treatment [14]. Although ALDH1 was vastly studied as a CSC marker in other solid tumors, it has been identified as a CSC marker in ovarian carcinoma in recent years [32]. In their study, dual positive cells of ALDH1 and another traditional ovarian CSC marker CD133 were isolated directly from human tumor to initiate tumor in mice, and these cells displayed enhanced angiogenesis and tumorigenicity like other CSCs [32]. Moreover, the patients with CD133(+)/ALDH1(+) tumor cells displayed reduced PFS and OS [32].

Distinct expression levels and patterns of ALDH1 in various human epithelial cancers and the corresponding normal tissues were explored [16, 37]. Unlike breast, lung or colon cancers, ovarian cancer displayed a significantly reduced ALDH1 expression compared to benign tumors and normal ovary [38], indicating a possibly different role of ALDH1 in ovarian cancer.

Our present immunohistochemical study of 248 well-characterized patients showed high levels of ALDH1 expression in ovarian carcinoma cells, which were observed in 15.7 % of the total cases, was associated with early-stage tumor, well-differentiated tumor and better survivals, although ALDH1 was not an independent risk factor in multivariate analysis. The patients involved in the current study were followed up for more than 12 years. To our best knowledge, it is a long follow up study, which has more predictive meanings in retrospective studies.

Our results were similar with a previous immunohistochemical study from The University of Texas MD Anderson Cancer Center. In their long-term follow-up study, The same mouse monoclonal anti-human ALDH Clone 44 from BD Transduction Laboratories™ was used, and it turned out that high level of ALDH1 was detected in 19 % out of the total 442 ovarian carcinoma samples, and it was significantly associated with endometrioid adenocarcinoma, early-stage tumor, complete response to chemotherapy, low serum CA125 level and favorable survivals [24]. Similar results were obtained by immunofluorenscence-based and quantitative approach in Rimm’s group from Yale University School of Medicine, a better prognosis in ALDH1(+) patients with non-small cell lung cancer, using the same primary antibody with us [39]. Thus, the choice of antibody may potentially explain the variably predictive and prognostic role of ALDH1 in human epithelial cancers. The current antibody we used has been proved specific and cytoplasmic with homogeneous staining pattern in different areas from the same case [39]. In this study, the homogeneously strongly positive cases (with total score 7 and 8) showed a significantly better survival probability.

However, there were other studies indicating a better clinical outcome in ALDH1(+) cancer patients, using different antibodies. Our previous study, using rabbit polyclonal antibody against human ALDH1A1 (ab63026, Abcam, Cambridge, UK) has proved that ALDH1 expression in vulvar squamous cell carcinomas predicted a significantly better survival than the ALDH1 negative cases [28]. Similarly, Hessman and coworkers, using antibody from Abcam, Cambridge, found that ALDH1 was highly expressed in early stage colorectal cancer in contrast with advanced stages [16].

It should be noted that ALDH1 in normal stem cells has a function of activating cell differentiation through retinoid acid signaling pathway, and the inhibition of ALDH1-mediated retinoid signaling impairs human fetal islet cell differentiation and survival [5]. It is also known that cancer stem cells share features of normal stem cells. Therefore, it can’t be excluded in the ovarian cancer cells that ALDH1 exerts its role through the same molecular mechanism, by such contributing to the better survival in ovarian cancers, although other unknown molecular mechanisms should be explored.

ALDH1 expression in stromal cells was previously reported to be frequently and strongly expressed in both non-malignant and tumor-associated stromal cells [28, 40–42], which was confirmed in our present study. However, the potential role of ALDH1 expression in the tumor microenvironment is rather different in the above findings. Resetkova et al. hold the opinion that high expression of ALDH1 in breast cancer stromal cells had a best disease free survival and a trend of better overall survival [42], De Brot et al. confirmed that ALDH1 frequent expression in tumor-associated stromal cells of triple negative breast cancer predicted a better outcome [41]. On the other hand, Woodward and Ohi together with their colleages insisted that ALDH1 expression in stromal cells of breast cancer was not associated to any clinical outcomes [43, 44]. In the present study, high expression level of ALDH1 in stromal cells was frequently observed in ovarian carcinoma, but the expression levels had no associations with the clinical parameters, and it is not associated with survival probabilities, which was in accordance with the findings from Woodward et al. [43] and Ohi et al. [44].

The current study has several limitations. First of all, although Allred scoring system combines the percentage and intensity of positive cells, as a manual scoring system, it may induce a level of subjectivity, especially the cut-off points were always a matter of discussion. Second, histological heterogeneity of ovarian cancers was not able to be addressed in the present study.

## Conclusion

In summary, the current views of ALDH1 predictive role in ovarian carcinomas remain controversial, and the present long-term follow-up retrospective study reveals that high ALDH1 expression in tumor cells portends favorable prognosis and better survivals in patients with ovarian carcinoma, but the expression of ALDH1 in stromal cells has no associations with clinical outcomes. More studies are warranted to verify the potential role of ALDH1 in ovarian carcinoma progression and the molecular mechanisms involved.
